# Achalasia in Pregnancy: Botulinum Toxin A Injection of Lower Esophageal Sphincter

**DOI:** 10.1155/2015/328970

**Published:** 2015-07-02

**Authors:** Nicole Hooft, Emily S. Schmidt, Ross M. Bremner

**Affiliations:** ^1^Norton Thoracic Institute, St. Joseph's Hospital and Medical Center, 350 W. Thomas Road, Phoenix, AZ 85013, USA; ^2^St. Joseph's Hospital and Medical Center, Creighton University School of Medicine, Phoenix Regional Campus, Phoenix, AZ, USA

## Abstract

*Background*. Achalasia, a rare esophageal motility disorder that may cause malnutrition during pregnancy, can result in fetal and maternal morbidity and mortality. Many medical treatment regimens are contraindicated or not tolerated during pregnancy, and surgery is generally avoided due to potential risks to the fetus. *Case Report*. Severe, medically refractory achalasia in a 23-year-old pregnant woman that caused malnutrition was successfully managed by administering a botulinum toxin A injection to the lower esophageal sphincter. The injection was performed at approximately 14 weeks' gestation and the patient reported clinically significant relief from dysphagia. She gained weight and ultimately delivered a healthy baby girl at term, but her symptoms returned a few months postpartum. She underwent a second treatment of botulinum toxin A injection, but it offered only one month of relief. Roughly eight months after delivery, the patient underwent a laparoscopic extended Heller myotomy and Dor fundoplication. The patient resumed a normal diet one week postoperatively, and her baby has had no complications. *Conclusion*. This is only the second reported case of botulinum toxin A injection being used to treat achalasia in pregnancy. This treatment proved to be a safe temporary alternative without the risks of surgery and anesthesia during pregnancy.

## 1. Introduction

Achalasia, a rare esophageal motility disorder, is characterized by a nonrelaxing lower esophageal sphincter (LES) and loss of peristalsis in the esophagus. Dysphagia is the most prominent symptom, and achalasia is occasionally associated with malnutrition syndromes. Both sexes are affected equally, and diagnosis typically occurs between the ages of 25 and 60 years [1]. Achalasia has an annual incidence of 1.6 in 100,000 persons and a prevalence of 10 in 100,000 persons [2], but achalasia during pregnancy is even rarer—only 12 cases have been reported in the literature [3]. In pregnant women, achalasia has been associated with maternal malnutrition and even death, preterm delivery, fetal growth restriction, and fetal death [4].

We report a case of achalasia during pregnancy, refractory to medical management, that was treated with botulinum toxin A injection to the LES. Only one other case has been reported of a patient treated during pregnancy with botulinum toxin A injection of the LES for this condition [5].

## 2. Case Presentation

A 23-year-old pregnant woman was referred to our institute with atypical chest pain, epigastric pain, and dysphagia. Her evaluation at the referring institution included a manometry study that showed diffuse esophageal spasm with a hypertensive LES and an esophagram that showed tertiary contractions with esophago-esophageal reflux and distal esophageal stricture. Evaluation for scleroderma and autoimmune disease was negative. Esophageal dilation was performed at the referring institution but did not resolve her symptoms. A trial of diltiazem was likewise unsuccessful. Imipramine and nitroglycerine had previously helped the patient somewhat, but her dysphagia continued to progress with further weight loss and a body mass index (BMI) of 17.5. The patient reported occasional alcohol use and had a remote social smoking history. The only medication that she used was cortisone cream, and she followed a self-restricted diet of soft foods.

**Figure 1:**
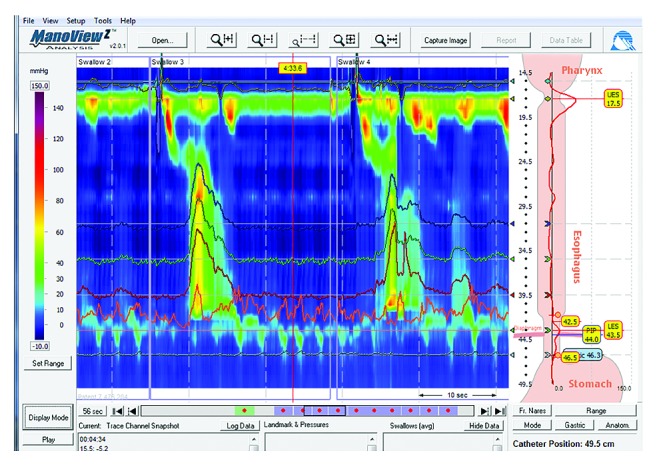
High-resolution manometry study showing hypertensive, nonrelaxing lower esophageal sphincter and aperistaltic esophagus, but with relatively high-amplitude simultaneous pressure changes in the esophageal body.

Upon presentation to our institute, the patient reported a continued weight loss of 1.3 kilograms over the preceding 5 weeks and her BMI was now 16.5. Her previous test results were reviewed and found to be consistent with type III achalasia (Figure 1). The patient had previously been scheduled for endoscopy, but when she discovered she was 7 weeks pregnant, she ceased all medications and canceled her planned endoscopy upon the advice of her obstetrician. Without treatment, her dysphagia worsened and she lost more weight, dropping to a BMI of 15.6. Her Eckardt score was 6 (dysphagia: 3, pain: 1, and regurgitation: 2), and she was treated for dehydration in the emergency department twice. Because medical management had failed to resolve her symptoms, surgery (Heller myotomy) and local botulinum toxin A injection to the LES during the second trimester were considered. The patient, her obstetrician, and our team chose to proceed with esophagogastroduodenoscopy (EGD) and botulinum toxin A injection and balloon dilation of the LES as a temporizing maneuver to help the patient through to term.

**Figure 2:**
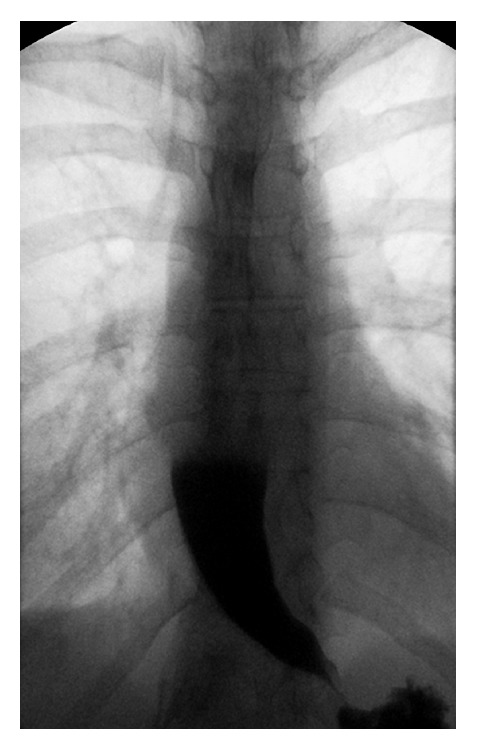
Esophagram showing classic “bird's beak” tapering with distal narrowing of the esophagus at the gastroesophageal junction with hang-up of contrast.

The procedure was performed at approximately 14 weeks' gestation. We injected a total of 100 IU/mL of botulinum toxin A in 4 mL of normal saline or 25 IU per aliquot per quadrant. Each aliquot was injected circumferentially into the submucosa just proximal to the LES. Balloon dilation of the LES was performed with a 20 mm balloon. After the procedure, the patient reported significant relief from dysphagia, and her Eckardt score improved to 3 (dysphagia: 1, pain: 1, and regurgitation: 1). She gained approximately 4.5 kilograms and her BMI increased from 16.5 to 18.2, which she maintained throughout the duration of her pregnancy. She ultimately delivered a healthy baby girl at term, but her symptoms returned a few months postpartum. She underwent a second treatment of botulinum toxin A injection and balloon dilation at her request (using the same dosage and balloon size as at the initial procedure), but the treatment provided only one month of relief. A subsequent barium esophagram revealed partially treated achalasia, with significant delay in esophageal emptying and a tapered lower esophagus (“bird's beak” sign) (Figure 2). Roughly eight months after delivery, the patient's Eckardt score returned to 6. She underwent a laparoscopic extended Heller myotomy and Dor fundoplication. The procedure was uneventful. The patient was able to resume a normal diet one week postoperatively, and she maintained her BMI of 18.2 postoperatively. Her baby has had no complications.

## 3. Discussion

Achalasia can be difficult to diagnose during pregnancy because it is rare and the symptoms are often attributed to gastroesophageal reflux disease, which many women experience while pregnant [3]. Once the diagnosis is made, however, numerous management strategies can improve the nutritional status of both the mother and the fetus without causing harm to either. Treatment options include diet modification, calcium channel blockers, nitrates, EGD with dilation, botulinum toxin A injection, total parenteral nutrition, Heller myotomy, and, in severe cases, percutaneous endoscopic gastrostomy (PEG) placement with enteral tube feedings [3]. Each of these interventions has potential risks, but laparoscopic surgical myotomy during pregnancy, in particular, is relatively contraindicated due to the potential harmful effects of anesthesia during pregnancy and the potential harm of a pneumoperitoneum and CO_2_ absorption to the fetus. No cases of harm to the fetus have been reported with the use of botulinum toxin A during pregnancy.

Botulinum toxin A injection is an attractive treatment option for pregnant patients because, unlike other medications used to treat achalasia, the injections have local rather than systemic effects. There have been no randomized controlled trials to study the effects of botulinum toxin A injections in pregnant patients, and these will likely never be performed. No long-term follow-up data exist on persons whose mothers received botulinum toxin A injections during pregnancy.

No research has been done on the efficacy of botulinum toxin A injection of the LES during pregnancy, and, to our knowledge, there is only one previously reported case of achalasia in pregnancy treated this way [5]. Injections of botulinum toxin A during pregnancy have been reported as a treatment for headaches, dystonia, spasticity, and for cosmetic purposes [6, 7], with no reported links to cases of fetal malformation or fetal demise. Some cases of spontaneous miscarriages in women treated with botulinum toxin A have been reported; however, each of these women also reported previous spontaneous miscarriages. The patients' histories of spontaneous miscarriage do not allow us to rule out injection of botulinum toxin A as the cause of miscarriage; however, the authors of these studies did not think that the botulinum toxin A was a contributing factor to the miscarriages [6, 7]. Botulinum toxin A remains a Pregnancy Category C drug; however, no trace of lethal doses injected into pregnant rabbits was detected in their fetuses [7]. There have also been 7 reported cases of wofmen who had botulism poisoning in their second or third trimesters, with no resulting birth defects or infantile botulism [7]. In fact, one of the cases of severe botulism poisoning described fetal movements as the only movements observed in the patient's body [8]. Because the achalasia in our patient was refractory to medical management and she continued to lose weight despite other treatments, we proceeded with the botulinum toxin A injection because the potential risks were outweighed by the risks of malnutrition to her and her fetus.

This report illustrates the safe treatment of recalcitrant achalasia with LES injection of botulinum toxin A. The effect was not long lasting, as is typical for this type of treatment, and our patient required surgical cardiomyotomy eight months after delivery. Botulinum toxin A injection of the LES proved to be a safe temporary alternative to surgery and the requisite anesthesia during pregnancy. In this case, the dysphagia was well palliated and the patient gained weight and delivered a healthy baby at term.
